# Humoral Immunity Links *Candida albicans* Infection and Celiac Disease

**DOI:** 10.1371/journal.pone.0121776

**Published:** 2015-03-20

**Authors:** Marion Corouge, Séverine Loridant, Chantal Fradin, Julia Salleron, Sébastien Damiens, Maria Dolores Moragues, Vianney Souplet, Thierry Jouault, Raymond Robert, Sylvain Dubucquoi, Boualem Sendid, Jean Fréderic Colombel, Daniel Poulain

**Affiliations:** 1 Université Lille Nord de France, Lille, France; 2 UDSL, Lille, France; 3 INSERM U995, Lille, France; 4 EA2694, Lille, France; 5 Pôle de Santé Publique Registre INSERM/InVS EPIMAD, Lille, France; 6 Service des Maladies de l’Appareil Digestif et de la Nutrition, Lille, France; 7 Service de Parasitologie Mycologie, Institut de Microbiologie, Lille, France; 8 CHRU, Lille, France; 9 Departamento de Enfermeria 1, Escuela Universitaria de Enfermeria, Universidad del Pais Vasco, 48940 Leioa, Spain; 10 Institut d’Immunologie, Pôle de Biologie, Lille, France; 11 Groupe d’Etude des Interactions Hôte-Pathogène, UPRES-EA 3142, Université d’Angers, Angers, France; 12 Innobiochips, 1 rue du Professeur Calmette, 59000 Lille, France; 13 Icahn School of Medicine at Mount Sinai, New York, NY, United States of America; King's College London Dental Institute, UNITED KINGDOM

## Abstract

**Objective:**

The protein Hwp1, expressed on the pathogenic phase of *Candida albicans*, presents sequence analogy with the gluten protein gliadin and is also a substrate for transglutaminase. This had led to the suggestion that *C*. *albicans* infection (CI) may be a triggering factor for Celiac disease (CeD) onset. We investigated cross-immune reactivity between CeD and CI.

**Methods:**

Serum IgG levels against recombinant Hwp1 and serological markers of CeD were measured in 87 CeD patients, 41 CI patients, and 98 healthy controls (HC). IgA and IgG were also measured in 20 individuals from each of these groups using microchips sensitized with 38 peptides designed from the N-terminal of Hwp1.

**Results:**

CI and CeD patients had higher levels of anti-Hwp1 (p=0.0005 and p=0.004) and anti-gliadin (p=0.002 and p=0.0009) antibodies than HC but there was no significant difference between CeD and CI patients. CeD and CI patients had higher levels of anti-transglutaminase IgA than HC (p=0.0001 and p=0.0039). During CI, the increase in anti-Hwp1 paralleled the increase in anti-gliadin antibodies. Microchip analysis showed that CeD patients were more reactive against some Hwp1 peptides than CI patients, and that some deamidated peptides were more reactive than their native analogs. Binding of IgG from CeD patients to Hwp1 peptides was inhibited by γIII gliadin peptides.

**Conclusions:**

Humoral cross-reactivity between Hwp1 and gliadin was observed during CeD and CI. Increased reactivity to Hwp1 deamidated peptide suggests that transglutaminase is involved in this interplay. These results support the hypothesis that CI may trigger CeD onset in genetically-susceptible individuals.

## Introduction

Celiac disease (CeD), also known as gluten-sensitive enteropathy, is a complex disorder where genetically-susceptible individuals develop signs of malabsorption and extra-intestinal manifestations after ingestion of cereals [1]. CeD affects 1% of the population of Europe and the USA, and can develop in early childhood or later in life [2]. The prevalence of CeD is considered to be underestimated [2].

The pathogenic mechanisms of CeD (schematized on S1 Fig. A) are induced by consumption of the cereals wheat, barley, and rye. These cereals contain a mixture of alcohol-soluble and alcohol-insoluble proteins known as gluten. Gliadins are a family of closely-related proline-rich and glutamine-rich alcohol-soluble proteins found in gluten. When peptides from dietary gliadin enter the sub-epithelial region they are deamidated by tissue transglutaminase [3]. Gliadin, deamidated gliadin, and transglutaminase are recognized by gliadin-reactive CD4+ T-cells in the *lamina propria* of individuals with HLA class II molecules DQ2 or DQ8 on antigen-presenting cells [4]. This results in a vigorous T-cell response that induces inflammation and tissue damage/remodeling (Th1 reaction). In parallel, a Th2 response is initiated which results in the production of IgG and IgA against exogenous gliadin [4] its deamidated form [5], as well as tissue transglutaminase [3]. The mainstay of treatment for CeD is a gluten-free diet (GFD). This results in a reduction of symptoms in parallel with a decrease in anti-gliadin and auto-anti-transglutaminase antibodies, and eventually histological healing [6]. It has become apparent that CeD, once believed to be primarily a disease of childhood, can develop in people of any age. The late appearance of CeD in genetically-predisposed individuals who have consumed gluten since childhood suggests the existence of an unknown trigger mechanism [7].

Human transglutaminases contribute to the maintenance of homeostasis through their activity on many endogenous physiologic substrates [8]. Their ability to interact with exogenous proteins is not limited to gliadin. Twelve years ago, Sundstrom *et al*. reported a process of molecular mimicry allowing the yeast *Candida albicans* to interact with transglutaminase [9]. *C*. *albicans* is a human commensal that colonizes all segments of the gastrointestinal tract and vagina of healthy individuals [10]. Epidemiologic and clinical studies in the past few decades have led to its recognition as an important public health problem in developed countries. The spectrum of diseases caused by this species ranges from vaginal infections, which affect up to 75% of women at least once in their lifetime[11] to invasive nosocomial infections, of endogenous origin, with high morbidity and mortality rates in severely ill patients [12]. It has been suggested that *C*. *albicans* may also play a role in the persistence or worsening of some chronic inflammatory bowel diseases through its ability to trigger the Th17 pathway [13]. Extensive and high quality research has concerned the pathophysiological mechanisms of host damage [14] and the genetic basis of host susceptibility [15]. From a microbial point of view, the success of *C*. *albicans* as a pathogen has been linked to its unique ability to take advantage of modifications to host homeostasis and microbiome alterations through highly sophisticated adaptative circuits [16]. Among the important factors contributing to pathogenesis is the ability of *C*. *albicans* to form germ tubes and hyphae leading to the expression of phase-specific adhesins and host tissue penetration [17]. The interaction between *C*. *albicans* and transglutaminase involves an antigen expressed specifically on the *C*. *albicans* germ tube cell wall, a protein named hyphal wall protein 1 (Hwp1). Covalent linkages established between human transglutaminase and Hwp1 enable *C*. *albicans* to bind strongly to human epithelial cells [9, 18, 19]. The Hwp1 amino acid sequences that act as a substrate for transglutaminase are identical or highly homologous to known CeD-related gliadin T-cell epitopes (S2 Fig.). These analogies have led to the hypothesis that *C*. *albicans* may be a trigger for CeD [20]. Although recent studies have reported an increased prevalence of anti-yeast mannan antibodies in patients with CeD [21–23], this hypothesis has not been explored further.

We developed a serological test for the detection of anti-Hwp1 antibodies in order to investigate the possible connection between *C*. *albicans* infection (CI) and CeD based on molecular mimicry between Hwp1 and gliadin. The serological responses against gliadin and Hwp1 were compared in patients with CeD and those with CI. The test was used in a cohort of CeD patients with different levels of compliance to a GFD, as well as in patients who developed CI during hospitalization, and in healthy controls (HC). The study of serological correlations between CeD and CI also concerned anti-transglutaminase and anti-deamidated gliadin antibodies [5, 24, 25]. Two different technologies were developed to assess the presence of anti-Hwp1 antibodies. The first consisted of an ELISA test involving recombinant Hwp1, and the second used microarrays sensitized with Hwp1 synthetic peptide analogs in their native or deamidated forms.

## Methods

### Patients and sera

For the first part of the study, 87 adults (median age: 35 years [range: 17–77]; 65 females) with CeD were included. Median time since diagnosis was 5 years [0–29]. Diagnosis of CeD was based on clinical, endoscopic, and histologic criteria. Twenty-eight patients had associated autoimmune disease, including four with selective IgA deficiency. The patients were divided into two groups according to their level of adherence to a GFD (strict or not strict adherence to a GFD in the previous 2 months).

Forty-six serum samples were obtained from 41 patients with systemic CI (median age: 58 years [12–85]; 22 males), hospitalized in Lille University Hospital. All patients were undergoing serological monitoring and had at least one *C*. *albicans*-positive blood culture. Longitudinal serum samples were obtained from five patients during the course of CI for kinetic analysis of anti-Hwp1 and anti-gliadin antibody responses. Ninety-eight serum samples from healthy blood donors served as negative controls (HC).

For the second part of the study, a second set of samples was used which consisted of 20 serum samples from patients with CeD (median age: 17 years [range: 1–57]; 14 females) including 14 samples from patients (11 females) without strict adherence to a GDF in the previous 2 months, 20 samples from new patients with proven systemic CI, collected at the time blood cultures were positive for *C*. *albicans* (median age: 61 years [30–79]; 11 males), and 20 additional sera from HC. All serological analyses were performed retrospectively from the residual frozen samples obtained from patients followed in the University Hospital of Lille.

### Ethics statement

All patients included in the study were followed in Lille University Hospital either in the Department of Gastroenterology (CeD patients) or Intensive Care Unit (CI patients). The care practices of both departments included serological testing to aid with diagnosis and therapeutic decisions. All complementary analyses designed to assess serological cross-reactivity between CeD and CI was made on residual sera anonymized and stored at -80°C in the clinical immunology laboratory and clinical mycology laboratory, respectively. No additional sample was made for the presented study.

As sera were taken from a registered biological collection bank (French Ministry of Research, reference DC-2008-642), patient consent was not required according to French law. Institutional review board approval was given by the “Comité de Protection des Personnes Nord-Ouest IV”, the ethical committee of our institution.

### Serological tests

#### Detection of serological markers of Celiac disease

Anti-gliadin IgG and IgA were detected using a commercially available ELISA kit (ELISIS Celiac Glia Check IgA/IgG; Biomedical Diagnostics) the results were expressed as optical density (OD). Anti-transglutaminase IgA and IgG were detected using a commercially available ELISA kit (Test Celikey TTA kit; Pharmacia Diagnostics), according to the manufacturer’s instructions. The results were expressed as arbitrary units (AU) with a positive cut-off of 8 AU/mL. IgG and IgA to deamidated gliadin were measured using an ELISA kit supplied by INOVA Diagnostics (Quanta Lite Celiac DPG Screen) with a positive cut-off of 20 AU/mL. Results were expressed using box-plots/scattered dots with AU values converted in logAU (see figures legends).

#### Development of tests for the detection of anti-Hwp1 antibodies

The study used a recombinant N-terminal non-glycosylated form of Hwp1 as described in S2 Fig. It was produced in *Escherichia coli*, a procedure previously shown to reveal antibodies specifically associated with CI [26]. This procedure was preferred to production in yeasts since a glycosylated form is likely to support cross-reactivity with anti-mannan antibodies [27].

The cloning, expression, purification and SDS-PAGE analysis of rNtermHwp1 are described in the S1 Materials and Methods A and B.

For characterization, we used the monoclonal antibody (mAb) 16B1, which binds to *C*. *albicans* hyphae expressing Hwp1 [28] and rNterm Hwp1 [29].

*ELISA*. Hwp1 antigen (0.3 μg), diluted in 0.01 M PBS, pH 7.4, was added to each well and incubated overnight at room temperature. The wells were then blocked by washing twice with bovine serum albumin and the plates were frozen. mAb 16B1 was diluted 1:1600 in TBST (0.05 M Tris HCl, pH 8.0, 0.15 M NaCl, 0.1% Tween 20) and revealed using horse radish peroxidase (HRP) conjugated anti-mouse IgGAM (Zymed) diluted 1:80 000. Human sera were diluted 1:50 and revealed with HRP conjugated anti-human IgG (Bio-Rad) using appropriate buffers and washing solutions (Bio-Rad). HRP conjugates were detected with TMB substrate (Promega) for 30 min at room temperature in the dark.

*Peptide arrays*. Experiments were performed with EpiFlag methodology (Innobiochips, Lille, France). Thirty-eight peptides of 20 amino acids each were designed from the Hwp1 sequence either as native overlapping peptides or with a switch of glutamine to glutamic acid in order to mimic the enzymic conversion carried out by transglutaminase. They were synthesized by solid phase peptide synthesis with an automated peptide synthesizer (Intavis AG, Köln, Germany) using the Fmoc/tert-butyl strategy and prepared, characterized, printed on glass slides, and probed with human or monoclonal antibodies as described in the S1 Materials and Methods C.

*Inhibition assays*. The peptides used in these assays are shown in S3 Fig. They consisted of: (i) sequences homologous to those designed from Hwp1 and used for sensitizing immunochips; (ii) sequences of 17–23 mers designed from different forms of gliadin: N1 as described by Anderson *et al*.[30], γ I and γ III as γ gliadin sequences presenting homologies with Hwp1 [20, 31] and (iii) non relevant peptide from mosquito saliva protein presenting no homology with Hwp1 and/or gliadin. Solutions of these peptides at concentrations of 10^-9^, 10^-7^, and 10^-5^ M were mixed with patients’ sera during incubation and the fluorescence signal was compared with that in their absence during the same run.

### Statistical analysis

All statistical analyses were performed using SAS version 9.1 (SAS Institute Inc., Cary, NC 25513). p values <0.05 were considered statistically significant.

Continuous variables are expressed as median [range]. Comparisons of antibody levels between HC, CeD patients, and CI patients were performed by analysis of variance (ANOVA) after rank transformation. Post-hoc analyses were done using Bonferroni correction. The interrelation between anti-Hwp1 antibodies and transglutaminase IgA and IgG, anti-gliadin IgA and IgG, and anti-deamidated gliadin IgA and IgG, and the association between changes in antibody titers and adherence to a GFD, were assessed using Spearman’s correlation coefficient. Comparisons according to GFD adherence were performed using the Mann-Whitney U test.

The results of peptide arrays are expressed as the percentage of sera from each group presenting signals considered as positive (> mean + 3SD of HC).

## Results

### Development of ELISA test to detect anti-rNtermHwp1 antibodies

**Fig 1:**
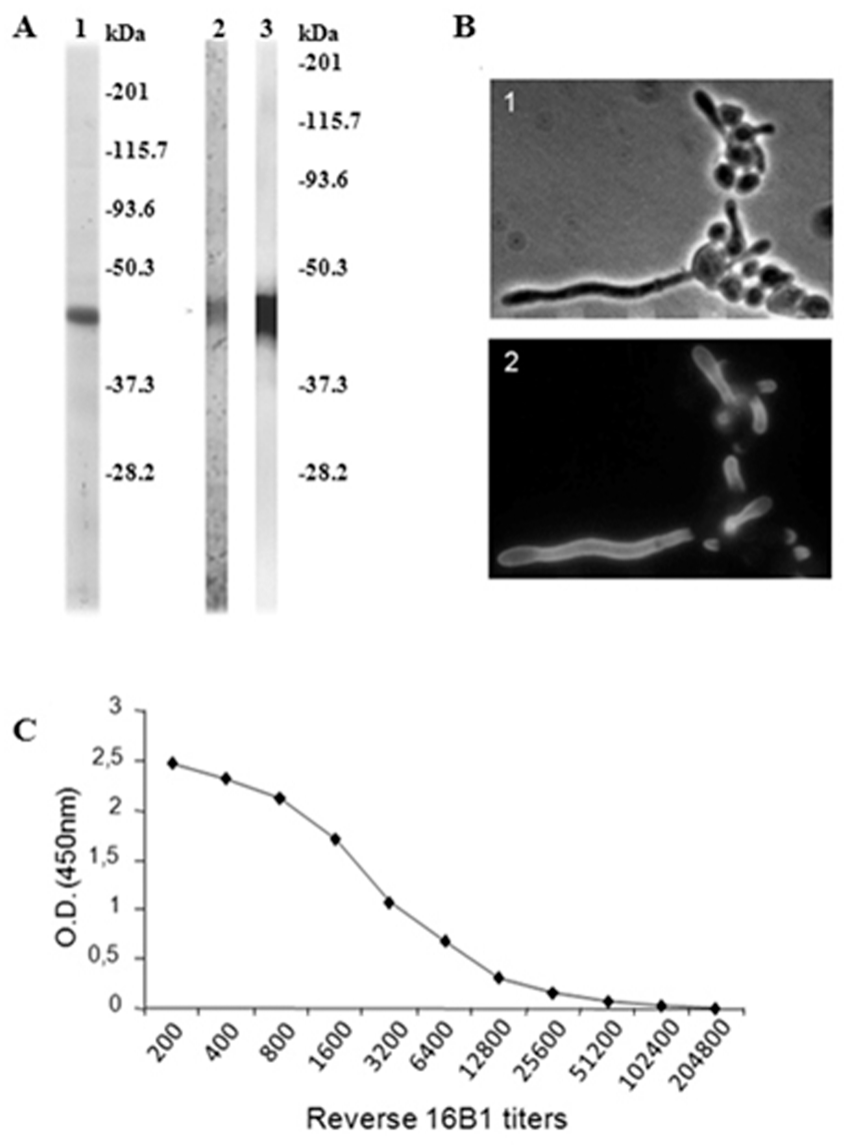
(A) Analysis of rNtermHwp1 purification by SDS-PAGE and immunoblotting. After migration in polyacrylamide gels, only a single band was stained with Coomassie blue in the sample containing purified rNtermHwp1 (lane 1). This band was detected with anti-HisTag (lane 2) and mAb 16B1 (lane 3) antibodies, showing purity of the sample. As deduced from its amino acid sequence, rNtermHwp1 has a theoretical molecular mass of 19.5 kDa but migrates in SDS PAGE as a 45 kDa protein. This molecular mass shift is a result of the rigid coiled structure of Hwp1. **(B) Photomicroscopy. (1)** Bright field microscopy of *C*. *albicans* yeast forms producing germ tubes. **(2)** Same microscopic field as **(1)** using immunofluorescence staining with mAb 16B1, which selectively binds to the surface of germ tubes. **(C) Reactivity of mAb 16B1 with rNterm Hwp1 by ELISA, expressed as optical density**.

As shown in Fig. 1, purification of rNterm Hwp1 resulted in a single band on PAGE (lane 1) which reacted in western blot with anti-HisTag antibodies (lane 2) used to purify the protein and mAb 16B1 (lane 3), specific for Hwp1 (Fig. 1B). This mAb selectively stains C. albicans germ tubes (Fig. 1A). This band had a relative molecular mass of 45 kDa instead of 19.5 kDa as predicted by its sequence (S2 Fig.). When this protein was used to coat ELISA plates, a typical dose-dependent curve was observed when mAb 16B1 was used as a standard (Fig. 1C).

### Anti-Hwp1 antibodies and serological markers of CeD in patients with CeD and those with CI

**Fig 2:**
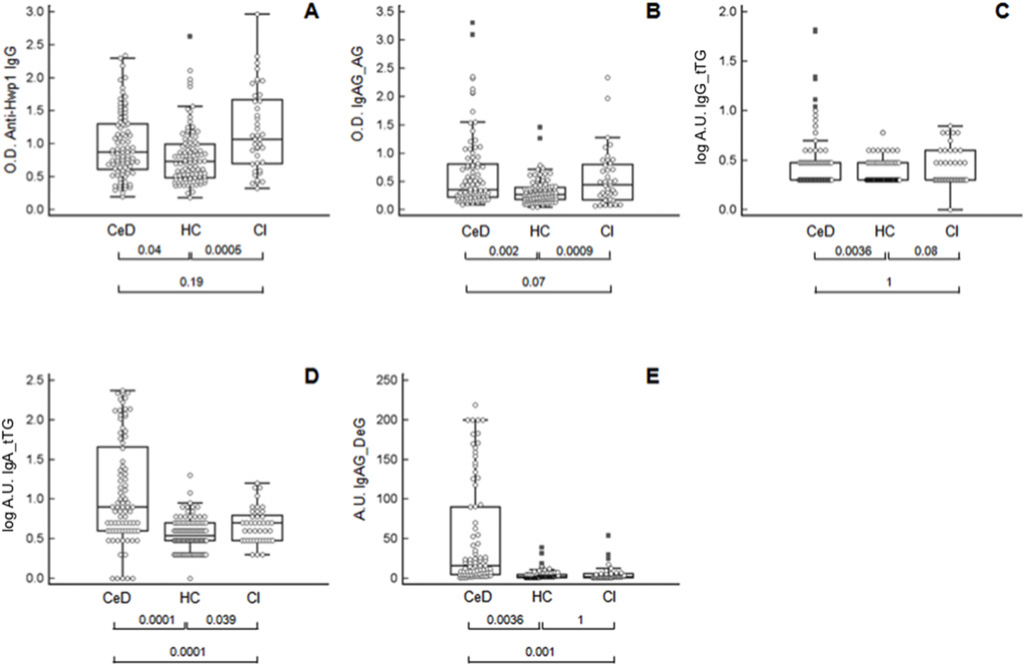
Distribution of antibodies in patients with Celiac disease (CeD), invasive *Candida* infection (CI), and healthy controls (HC). **(A)** Anti-Hwp1 IgG; **(B)** anti-gliadin IgA and IgG; **(C)** anti-transglutaminase IgG; **(D)** anti-transglutaminase IgA; **(E)** anti-deamidated gliadin IgA and IgG. Anti-Hwp1 IgG, anti-gliadin IgA and IgG results are expressed as optical density (450 nm), and anti-transglutaminase IgG and IgA **as log of arbitrary units (log AU)** and anti-deamidated IgA and IgG results as arbitrary units (AU). The significance of discrimination between the different groups is represented below each figure as the *p* value.

Fig. 2 shows the results of antibody detection tests performed on sera from patients with CeD and CI in comparison to HC. Antibodies against Hwp1 (Fig. 2A) discriminated between patients with CI and HC (p = 0.0005). CeD patients also had significantly higher levels of anti-Hwp1 antibodies than HC (p = 0.04), but anti-Hwp1 antibody levels did not discriminate between CI and CeD (p = 0.19). Anti-gliadin antibodies (Fig. 2B) discriminated between CeD patients and HC (p = 0.002), and also discriminated patients with CI from HC (p = 0.0009). Anti-gliadin antibodies did not discriminate between CeD and CI (p = 0.07).

Among the other serological markers of CeD, anti-transglutaminase IgA and IgG (Fig. 2C and D) discriminated CeD from HC. Both isotypes were increased in CI, without reaching significance for IgG. A comparison of CI and CeD showed that IgA levels were higher in CeD whereas IgG levels were the same in both diseases. Finally, anti-deamidated gliadin IgA and IgG (Fig. 2E) were the only antibodies specific for CeD. These antibodies could discriminate CeD from both HC and CI, which gave similar results.

When the correlation between anti-Hwp1 antibodies and serological markers of CeD was assessed (Table 1), a significant correlation with anti-gliadin was found in both CeD and CI (p = 0.002 and p = 0.01, respectively).

**Table 1:** Significance (p values) of the correlation between anti-Hwp1 IgG and markers of Celiac Disease (CeD) in CeD patients and patients with *Candida albicans* infection (CI) (COOR procedure, Spearman’s test).

	Anti-gliadin IgA and IgG	Anti- transglutaminase IgA	Anti-transglutaminase IgG	Anti-deamidated gliadin IgA and IgG
CeD patients	**0.002**	**0.004**	0.13	**0.02**
CI patients	**0.01**	0.1	**0.01**	0.4

Significant values are shown in bold.

### Anti-Hwp1 and anti-gliadin antibodies during the course of *C*. *albicans* infection

**Fig 3:**
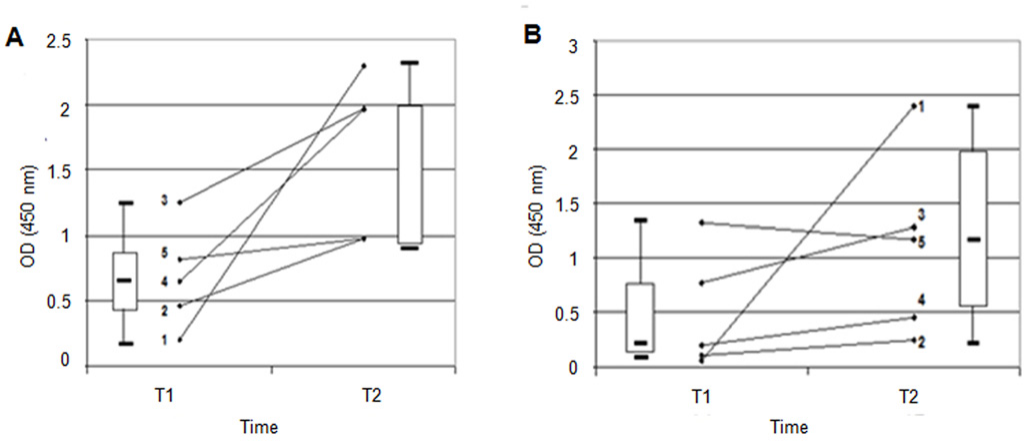
Kinetics of anti-Hwp1 and anti-gliadin antibody responses. **(A)** Anti-Hwp1 and **(B)** anti-gliadin antibodies in five patients with invasive *C*. *albicans* infection (CI) selected for having an increase in anti-Hwp1 IgG during infection. Each number represents a patient and the results are expressed as optical density. The anti-Hwp1 response parallels the anti-gliadin response (Box Plots) except in one patient (no. 5).

The dynamics of this correlation were investigated by selecting CI patients who had an increase in anti-Hwp1 antibodies during the course of their infection (Fig. 3A). An increase in anti-gliadin antibodies was also observed in the same sera (Fig. 3B). With the exception of one patient (no. 5), who had only a small increase in anti-Hwp1 antibodies, all other patients had an increase in anti-gliadin antibodies that paralleled the anti-Hwp1 response. Data from patient 1 were particularly interesting since both anti-Hwp1 and anti-gliadin levels were negligible at the onset of infection but increased during the development of CI. When CI was established, anti-gliadin antibodies reached levels considered as highly significant for CeD.

### Serological markers of CeD and anti-Hwp1 antibodies according to compliance to a gluten-free diet

**Fig 4:**
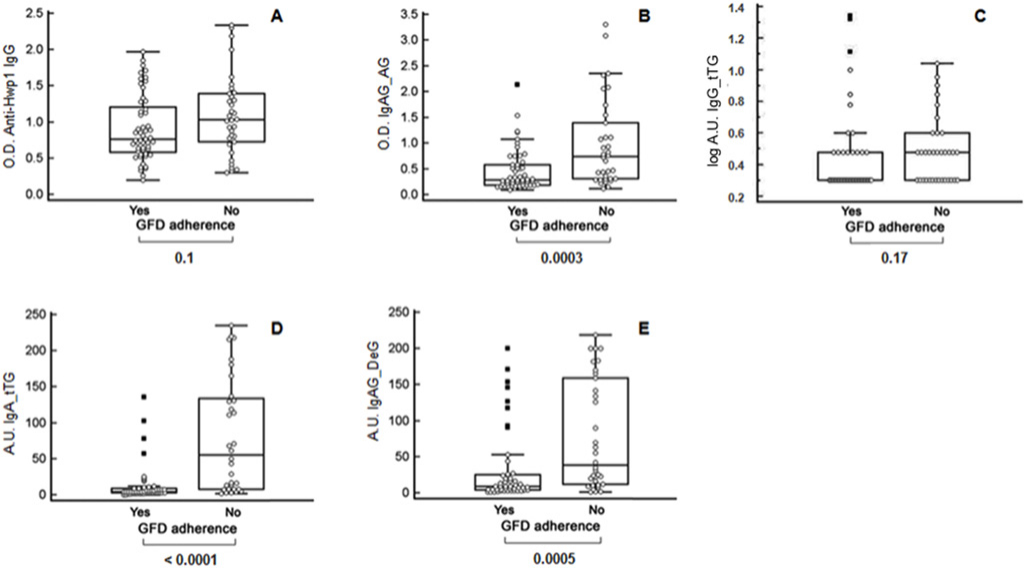
Antibody reactivity of sera from Celiac disease (CeD) patients according to adherence to a gluten-free diet (GFD). **(A)** Anti-Hwp1 IgG; **(B)** anti-gliadin IgA and IgG; **(C)** anti-transglutaminase IgG; **(D)** anti-transglutaminase IgA; **(E)** anti-deamidated gliadin IgA and IgG. Anti-Hwp1 IgG, anti-gliadin IgA and IgG results are expressed as optical density (450 nm) anti-tTG IgG as log of arbitrary units (log AU) anti-IgA tTG and anti-deamidated gliadin as arbitrary units (AU). YES: strict adherence to a GFD in the previous 2 months. NO: no strict adherence in the previous 2 months.

When CeD patients were classified into two categories according to GFD compliance (Fig. 4), those who were compliant had significantly lower levels of anti-gliadin and anti-transglutaminase IgA CeD biomarkers than non-compliant patients (Fig. 4B, D and E). In contrast, the differences observed for anti-Hwp1 IgG and anti-transglutaminase IgG (Fig. 4A and C) did not reach significance.

### Serological studies involving microchips sensitized with synthetic homologs of Hwp1 peptides

**Fig 5:**
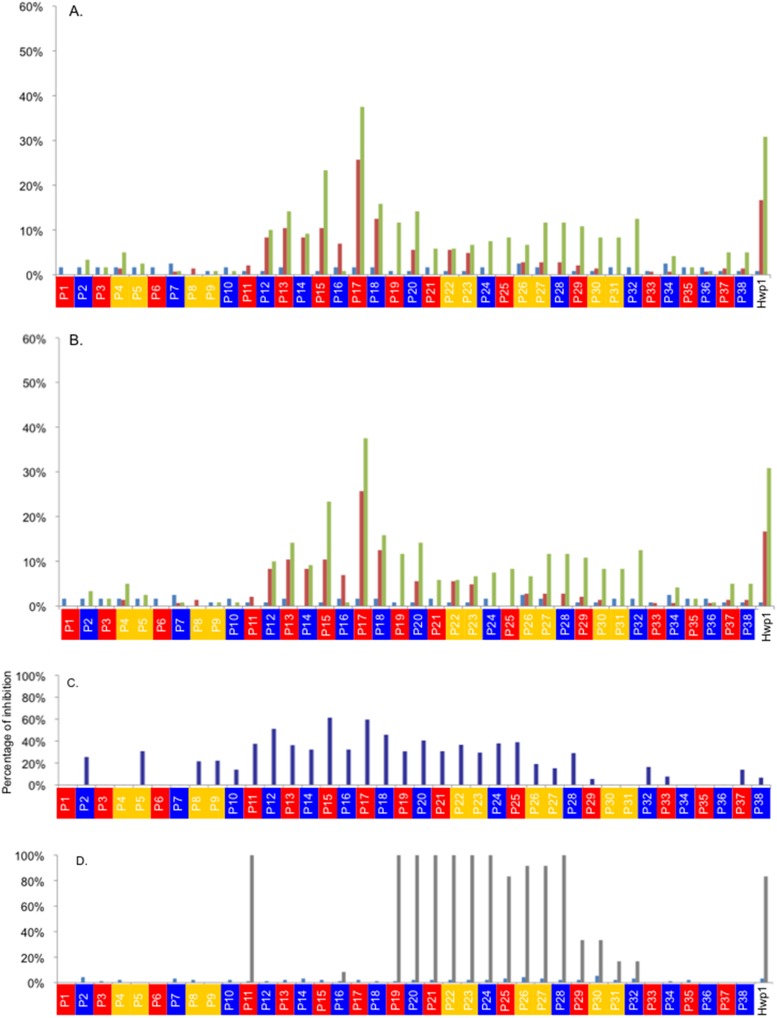
Percentage of sera giving positive IgA (A) and IgG (B) signals, respectively, on the 38 synthetic peptides (with color code according to their design*) as a function of the nature of the sera (CeD in green, CI in red, and HC in blue). * Red: native peptide; blue all Q changed for E; yellow: combinatory changes for one Q/E (Glutamine/Glutamic acid). (**C)** Reduction of signal resulting from the addition of 10^-5^ M of a 20-mer sequence mimicking γ gliadin (γ III) to different Hwp1 peptides. **(D)** Nature of Hwp1 peptides reacting with mAb 16B1 (epitope mapping) (HC in blue, Ac16B1 in grey).

Thirty-eight overlapping 20-mer peptides were designed from Hwp1 N-term and in some of them glutamine was replaced by glutamic acid in order to mimic deamidation. rNterm Hwp1 was used as a control. The sequences of these peptides are shown in S3 Fig. The results obtained for the detection of IgA and IgG to these peptides in CeD, CI, and HC sera are shown in Fig. 5A and B. Low reactivity was observed for all HC sera. Interestingly, more CeD patients reacted with peptides mimicking the *C*. *albicans* protein Hwp1 than CI patients; a similar observation was made for Hwp1 rNterm. Reactivity in both diseases was concentrated on peptides 11–21. P17 displayed the highest reactivity against both CeD and CI sera; this reactivity was even higher than that of Hwp1 Nterm. Partly deamidated P17 maintained this reactivity. Interestingly, for weakly reactive P11, which contained seven glutamine residues, replacement of glutamine by glutamic acid resulted in the appearance of reactivity with some sera from CeD patients. This demonstrates that this process mimicking transglutaminase activity on Hwp1 induced increased recognition by CeD sera exhibiting high levels of anti-deamidated gliadin. Fig. 5D shows that the epitopes recognized by anti-Hwp1 mAb 16B1 generated in mice and used as a reference did not map in the same area as the epitopes recognized by patient immunoglobulins.

### Evidence for cross-reactivity in CeD between Hwp1 and gliadin peptides

**Fig 6:**
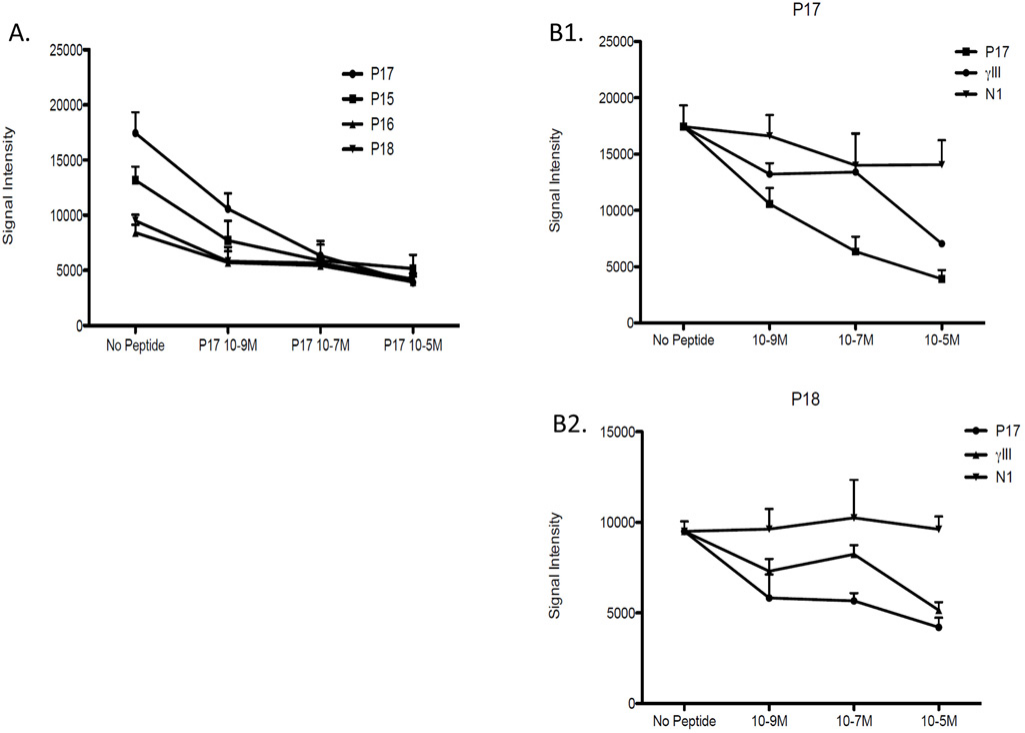
(A) Reduction of fluorescent signal associated with the binding of patient’s IgG to P17 as a function of different molarities of neighboring overlapping peptides P15,16,18 (deamided or not) (B1, B2). Representative examples of inhibition of CeD serum reactivity to P17 and P18 using the homologous peptides and N1 and γ III gliadin sequences.

Inhibition reactions were performed using sera from CeD patients with high levels of IgG reactivity against Hwp1 peptides. Special attention focused on peptides 11–18 against which reactivity was observed in both CeD and CI patients and which displayed maximum inhibition in presence of 10^-5^ M of a 20-mer sequence mimicking γ gliadin (γ III) to different Hwp1 peptides (Fig. 5C). When considering homologous inhibition as a positive control, a typical dose-dependent curve was observed when different concentrations of P17 were used to inhibit IgG reactivity against the same peptide and with P15, 17, 18 as overlapping neighboring sequences (deamidated or not) (Fig. 6A). Inhibition reactions involving all these peptides resulted in a typical dose-dependent curve with a maximal slope for the homologous peptide P17. Fig. 6 B1 and B2 shows representative examples of inhibition of CeD sera reactivity against Hwp1 peptides using peptides designed from gliadin sequences. For both P17 and P18, the N1 sequence did not result in any inhibition in contrast to the sequence from γ III which inhibited reactivity of CeD sera with the Hwp1 peptide. Like inhibition with homologous fungal peptides this was dependent on molarity, providing definitive evidence of cross-reactivity between Hwp1 and gliadin. When considering the percentage inhibition resulting from addition of γ III at a maximal concentration of 10^-5^ M to whole microchips, a Gaussian pattern was observed which superimposed the Hwp1 peptide area where maximum cross-reactivity was observed between sera from CeD and CI patients.

## Discussion

Our results using recombinant Hwp1 in ELISA demonstrate, for the first time, serological cross-reactivity in humans between this *C*. *albicans* antigen and gliadin, confirming a hypothesis proposed several years ago suggesting that sequence homologies between these two antigens can lead to cross-immune recognition [20]. This demonstration involved sera from patients with CeD and CI and considers that the Th2 response parallels the Th1 response in causing tissue damage and related symptoms [3]. However, we encountered difficulties in the preservation of Hwp1 relevant CeD epitopes for ELISA development. These difficulties, which probably explain why this hypothesis has not been investigated previously, could be attributed to the rigid coiled structure of Hwp1 altering its immunologic reactivity, as well as its high proline content (27%)[19] (a character shared with gliadin), and finally, a dipeptide composition classifying the molecule as unstable [32]. The molecule was produced in *E*. *coli*, in order to avoid interference due to antibodies against its polysaccharide moiety when produced in yeast [27]. Controls performed using this recombinant protein in an ELISA test showed its ability to discriminate between serological responses of CI patients and HC in agreement with its role in invasion, including its activity as a substrate for mammalian transglutaminase to establish covalent links between germ tubes and epithelial cells [9, 18]. Subsequent microarray studies confirmed the results of ELISA and showed that sera from CI patients reacted at different levels to HC sera with synthetic peptides designed from Hwp1, even those that mimicked hypothetical Hwp1 deamidated forms. Using this methodology, dose-dependent inhibition was observed.

This study was not intended to re-assess the diagnostic value of antibody detection tests to Hwp1 for diagnosis of CI [26]; neither the one of anti-gliadin and anti-transglutaminase antibody detection tests for the diagnosis of CeD-not used any longer due to low specificity. By contrast, our aims focused on analyzing basic correlations between antibody responses against different antigens in two diseases by using sera from with clinically confirmed diseases through biopsies for CeD patients and blood cultures for CI patients and careful analysis of clinical files. Incidentally it is of note, on a diagnostic point of view, that the correlations evidenced would account for false positives in both CI and CeD serological tests.

ELISA revealed that anti-Hwp1 IgG titers were increased in patients with CI when compared to HC, but did not discriminate between CI and CeD. Conversely, anti-gliadin antibodies were significantly associated with CeD compared to HC, but did not discriminate between CeD and CI. Strikingly, there was no significant difference in antibody levels against either Hwp1 or gliadin between CeD and CI patients. The correlation observed between anti-Hwp1 and anti-gliadin antibodies (Table 1) further supports the observation that anti-Hwp1 antibody levels are linked to anti-gliadin antibody levels during both CI and CeD. This suggests that human antigen-presenting cells/T-cells may induce cross-reactivity towards both processed antigens in the two pathologic circumstances represented by CeD and CI. Additional evidence was obtained from sera obtained sequentially from patients during CI. In patients who produced an IgG response against Hwp1 following CI, anti-gliadin levels increased in parallel. Together, these data suggest that fungal Hwp1 protein may be recognized by the human immune system in a similar way to gliadin, which induces CeD in genetically-predisposed individuals with the HLADQ2/8 genotype [33]. The correlation with Hwp1 rNterm did not concern the 10-mer sequence mimicking deamidated gliadin [4]. However, when using Hwp1 amino acid sequences designed for microchip analysis some Hwp1 20-mer peptides constructed with glutamic acid instead of glutamine were more reactive than their native analogs against some sera from CI and CeD patients. This suggests that mimicking the conversion established *in vivo* by transglutaminase affected the immune reactivity against both gliadin and Hwp1, reinforcing the pathogenic potential of this cross-reactivity.

The presence of anti-yeast antibodies (known as ASCA) has been previously reported in CeD leading to the suggestion that microbial targets may have a role in the early development of celiac disease whereas previous studies interpreted these results as a consequence of increased intestinal permeability [20, 22, 34]. Investigations in patients with Down’s syndrome concluded that the presence of ASCA was related more to alterations in the induction and/or maintenance of tolerance of microbes than to increased intestinal permeability [35]. In contrast with anti-fungal glycan antibodies, like ASCA, anti-Hwp1 antibodies reflect the saprophytic-pathogenic transition of *C*. *albicans* independent of intestinal integrity [26, 36]

The decrease in levels of anti-gliadin antibodies in GFD-adherent compared to non-adherent CeD patients further validates the clinical classification used. In contrast, the relative independence of anti-Hwp1 antibodies from a GFD suggests the involvement of different subsets of memory cells, and that once the disease is triggered, possibly through CI by cross-reactivity between *C*. *albicans* Hwp1 and gliadin, gliadin then becomes the predominant antigen due to its abundance in the diet and continuous stimulation [1] of the immune system.

In addition to the measurement of anti-Hwp1, anti-gliadin, and anti-deamidated gliadin antibodies in patients with CI and CeD, our cross-analysis also included the detection of anti-transglutaminase antibodies. The increase in anti-transglutaminase antibodies observed in CeD patients compared to HC paralleled that observed with anti-gliadin in accordance with the hypothesis that both antigens could be presented to T-lymphocytes as a complex processed by dendritic cells [3]. We therefore asked the question: “could *Candida* infection induce an increase in anti-transglutaminase antibodies?” considering that in a non-genetically-susceptible individual such an auto-immune process would be down-regulated by elimination of auto-reactive lymphocytes. It is interesting to note that active transglutaminase was recently shown to be expressed on dendritic cell membranes [37] suggesting that the close contact established with *C*. *albicans* hyphae during infection[38] places the enzyme in direct contact with Hwp1. Indeed, anti-transglutaminase levels were lower in CI than in CeD patients, but patients with CI nevertheless presented significantly higher anti-transglutaminase IgA levels than HC, whereas the differences in anti-transglutaminase IgG were close to the limit of significance. Interestingly, a significant correlation was observed between anti-transglutaminase IgG and Hwp1 in CeD patients suggesting that the increase in anti-Hwp1 IgG during CI is associated with a moderate immune response against this host enzyme.

This study provides data establishing a serological link between CI and CeD. Other studies have reported the presence of anti-gliadin antibodies in patients with autoimmune polyendocrine syndrome type I [39] and chronic mucocutaneous candidiasis (CMC) [40, 41]. CMC is characterized by chronic *Candida* infection of the skin, mucous membranes and nails where extensive vegetations of *C*. *albicans*, including hyphal forms expressing Hwp1, are found. Garcia *et al*. [41] suggested that the presence of anti-gliadin antibodies in CMC was related to the underlying immunologic disorder. However, the dramatic decrease in anti-gliadin antibodies titers observed in the study of Brinkert *et al*.[40] after successful treatment of CI also supports the theory of cross-reactivity between anti-gliadin and anti-Hwp1 antibodies [20]. It is therefore possible that, through a possible overlap in immune recognition of Hwp1 and gliadin, CI could trigger the onset of CeD in genetically-susceptible individuals (S1 Fig. B and C). Attention should probably be raised if there is suspicion of CI and onset of gluten intolerance. For example antibiotic therapy is long recognized as a major risk factor for tissue invasion, since oral and systemically delivered antibiotics promote *C*. *albicans* proliferation through elimination of subsets of microbiota [42]. As described for the identification of gliadin prominent peptides [43], it would be worthwhile to explore T-cell reactivity against Hwp1-derived peptides, particularly in view of evidence from dose-dependent inhibition reactions between peptides from both origins using CeD patient immunoglobulins. In addition, the results from microchip analysis of native or deamidated Hwp1 peptides provide interesting data to explore the finely tuned mechanisms of such recognition

Interestingly, anti Hwp1 and anti-gliadin antibodies were observed in the different groups of patients and controls—through at low level- suggesting that they may occur as natural autoantibodies. One hypothesis is that these antibodies which are important for immune homeostasis, may undergo somatic hypermutation and turn into disease inducing antibodies under the influence of a pathogen [44, 45]. Conversely, evidence for no impact of the timing of introduction of gluten in the diet on the occurrence of the disease still recently focuses attention on missing environmental factors [46].

Research on CeD has led to the current evolution of ideas regarding the role of intestinal antibody-secreting cells [47] and role of post-translational modifications of antigens to break T-cell tolerance to self-antigens and promote autoimmune disease. Although neglected by bacteriologists, the pathogenic transition of *C*. *albicans* is pathognomonic of changes in the microbiota [42]. As such, it shares characteristics of an exogenous driver of post-translational modifications [48]. This study has revealed immune cross-recognition between two substrates of the potential auto-antigen transglutaminase, namely the fungal “invasive” protein Hwp1 and the dietary ‘innocuous” vegetal protein gliadin. Together our data, obtained from a translational comparative analysis of an infectious and an auto-immune disease, support the hypothesis [20] that the former may trigger the development of the latter.

## Supporting Information

S1 Fig (A) Pathogenesis of celiac disease (adapted from Schuppan *et al*. [3]) Infections or mechanical or chemical injury, which lead to an increased permeability of the mucosal epithelium, facilitate the entrance of dietary gluten peptides into sub-epithelial regions. In the lamina propria, the gluten peptides encounter TG2 which is released from stressed endothelia, fibroblasts, and inflammatory cells residing in the subepithelial region. Crosslinking of gluten by TG2 potentiates its uptake and presentation by antigen-presenting cells, and its deamidation improves the binding to HLA-DQ2/8 molecules. The presentation of the gluten peptides on professional antigen-presenting cells, in particular dendritic cells, triggers a vigorous T cell response that induces inflammation and tissue remodeling (Th1 reaction) or antibody production (Th2 reaction). **(B) The role of Hwp1 during a *C*.*albicans* infection**. Under certain conditions, such as antibiotic treatments, *C albicans* can damage the intestinal epithelial cell barrier, thus raising extracellular concentrations of tissue transglutaminase that link covalently to Hwp1 expressed at the cell wall surface of *C albicans* invasive hyphae. *C albicans* molecules among which are Hwp1 complexed with transglutaminase will be processed by antigen-presenting dendritic cells locally recruited/activated at the site of infection and presented to T cells. **(C) Contribution of the study in linking events together (Arrows)**. Among the consequences of this T cell activation is the specific development of B cell lines with a IgG repertoire refined for anti-Hwp1 antibodies. The results gained in this study strongly suggest (arrow of Part C: linking parts A and B) that anti-Hwp1 IgG can react with gliadin. This observation is compatible with the hypothesis that initiation of recognition and then amplification of the response towards this T cell molecule could concern gliadin as well, a process which in HLADQ2-DQ8 genetic background is liable to trigger gluten intolerance at any age of life.
(TIF)

S2 Fig Homology of *C*. *albicans* Hwp1 with gliadin and T-cell gliadin epitopes. **(A)** Amino acid sequence alignment of Hwp1 and one member (Genbank: ACM41414.1) of the common wheat Triticium aestivum subsp. Macha, α gliadin family. The rNtermHwp1 sequence is framed and the rHwp1T sequence is shown below the solid line. The known transglutaminase substrate sequence includes amino acids 41–197. The T-cell epitope of γ gliadin is shown in bold. Identity between the two sequences is shown in red. **(B)** Multiple amino acid sequence alignment of rNtermHwp1 and different T-cell gliadin epitopes. T-cell epitopes from different members of the γ-gliadin (1: DQ2-g-III; 2: DQ2-g-IV; 3: DQ2-g-V and γ-III plus; 4: g-1; 5–6: DQ2) and α-gliadin (7–9: DQ2) family are homologous to Hwp1. Identity between rNtermHwp1 and α-gliadins is shown in red. The clustalW2 program (http://www.ebi.ac.uk/Tools/clustalw2/index.html) was used for both alignments.
(TIF)

S3 Fig (A) Sequences of the 38 overlapping 20-mer peptides designed from Hwp1 N-term with color code according to their design. Native peptide (red); peptides with a switch of glutamine to glutamic acid in order to mimic the enzymic conversion carried out by transglutaminase: all Q changed for E (blue), combinatory changes for one Q/E replacement (yellow). Gray represents peptides not synthesized. Overlaps are underlined in black. **(B)** Sequences of the 17–23-mer peptides designed from different forms of gliadin (N1, γ I, γ III) and non-relevant peptide (NR). Homology motifs between Hwp1 and gliadin are in bold (PQQPQ: 3 and 5 repeating motif, respectively, in γ-gliadin and Hwp1; YPQQPQ, PQQQ: common motifs in γ-gliadin and Hwp1; PQPQLPY: α-gliadin motif having high sequence homology with the repeated sequence PQPDIPC in Hwp1).
(TIF)

S1 Materials and Methods (A) Description of Cloning, expression and purification of rNtermHwp1 (B) SDS-PAGE and western blotting (C) Peptide arrays. (DOCX)
