# Supplementary figures and images for: Humoral Immunity Links Candida albicans Infection and Celiac Disease
Source: PLoS One. 2015 Mar 20;10(3):e0121776. doi: 10.1371/journal.pone.0121776 (PMC4368562; doi:10.1371/journal.pone.0121776)

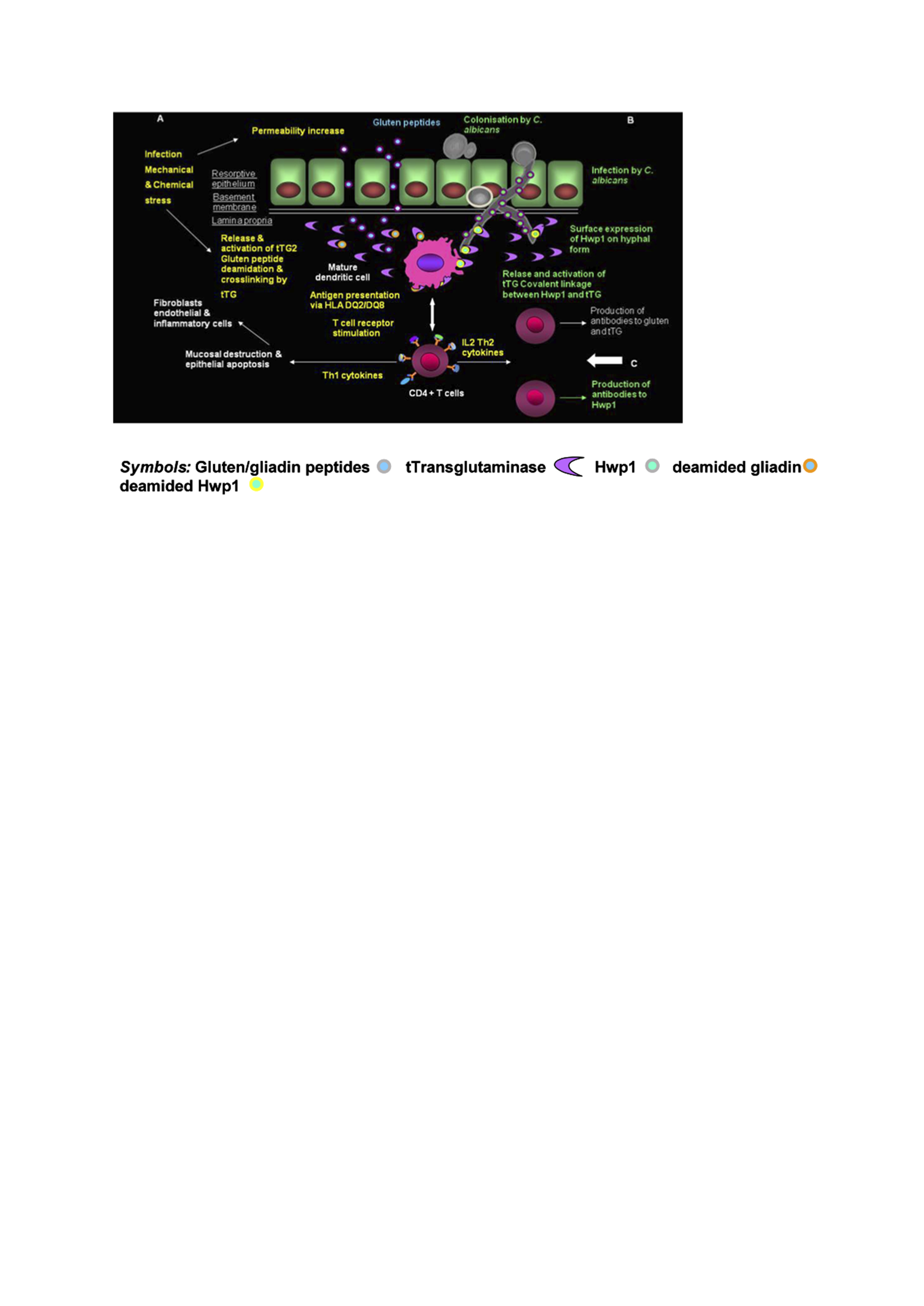

Supplement: S1 Fig — Infections or mechanical or chemical injury, which lead to an increased permeability of the mucosal epithelium, facilitate the entrance of dietary gluten peptides into sub-epithelial regions. In the lamina propria, the gluten peptides encounter TG2 which is released from stressed endothelia, fibroblasts, and inflammatory cells residing in the subepithelial region. Crosslinking of gluten by TG2 potentiates its uptake and presentation by antigen-presenting cells, and its deamidation improves the binding to HLA-DQ2/8 molecules. The presentation of the gluten peptides on professional antigen-presenting cells, in particular dendritic cells, triggers a vigorous T cell response that induces inflammation and tissue remodeling (Th1 reaction) or antibody production (Th2 reaction). (B) The role of Hwp1 during a C.albicans infection. Under certain conditions, such as antibiotic treatments, C albicans can damage the intestinal epithelial cell barrier, thus raising extracellular concentrations of tissue transglutaminase that link covalently to Hwp1 expressed at the cell wall surface of C albicans invasive hyphae. C albicans molecules among which are Hwp1 complexed with transglutaminase will be processed by antigen-presenting dendritic cells locally recruited/activated at the site of infection and presented to T cells. (C) Contribution of the study in linking events together (Arrows). Among the consequences of this T cell activation is the specific development of B cell lines with a IgG repertoire refined for anti-Hwp1 antibodies. The results gained in this study strongly suggest (arrow of Part C: linking parts A and B) that anti-Hwp1 IgG can react with gliadin. This observation is compatible with the hypothesis that initiation of recognition and then amplification of the response towards this T cell molecule could concern gliadin as well, a process which in HLADQ2-DQ8 genetic background is liable to trigger gluten intolerance at any age of life. (TIF) [file pone.0121776.s001.tif]

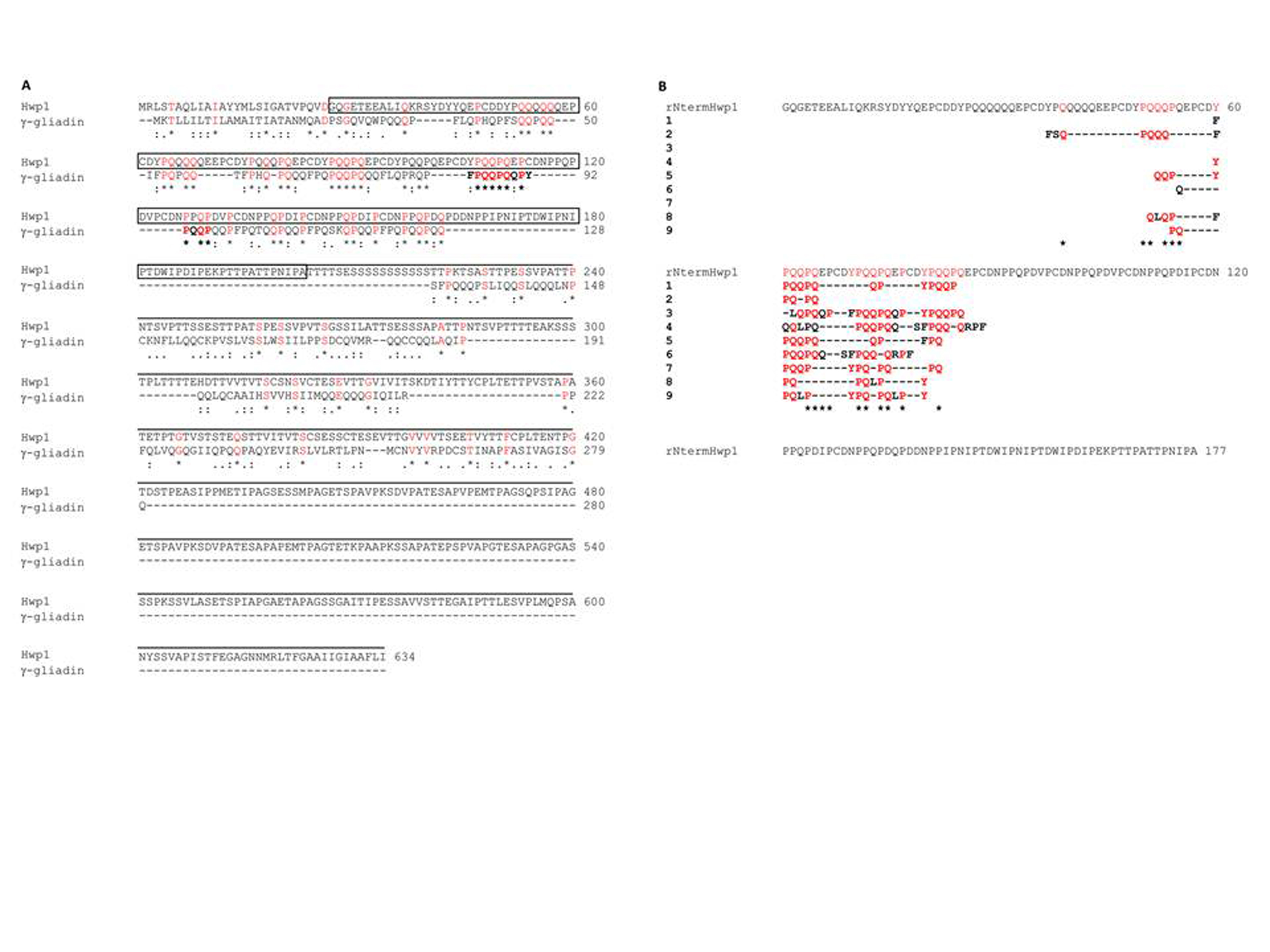

Supplement: S2 Fig — (A) Amino acid sequence alignment of Hwp1 and one member (Genbank: ACM41414.1) of the common wheat Triticium aestivum subsp. Macha, α gliadin family. The rNtermHwp1 sequence is framed and the rHwp1T sequence is shown below the solid line. The known transglutaminase substrate sequence includes amino acids 41–197. The T-cell epitope of γ gliadin is shown in bold. Identity between the two sequences is shown in red. (B) Multiple amino acid sequence alignment of rNtermHwp1 and different T-cell gliadin epitopes. T-cell epitopes from different members of the γ-gliadin (1: DQ2-g-III; 2: DQ2-g-IV; 3: DQ2-g-V and γ-III plus; 4: g-1; 5–6: DQ2) and α-gliadin (7–9: DQ2) family are homologous to Hwp1. Identity between rNtermHwp1 and α-gliadins is shown in red. The clustalW2 program (http://www.ebi.ac.uk/Tools/clustalw2/index.html) was used for both alignments. (TIF) [file pone.0121776.s002.tif]

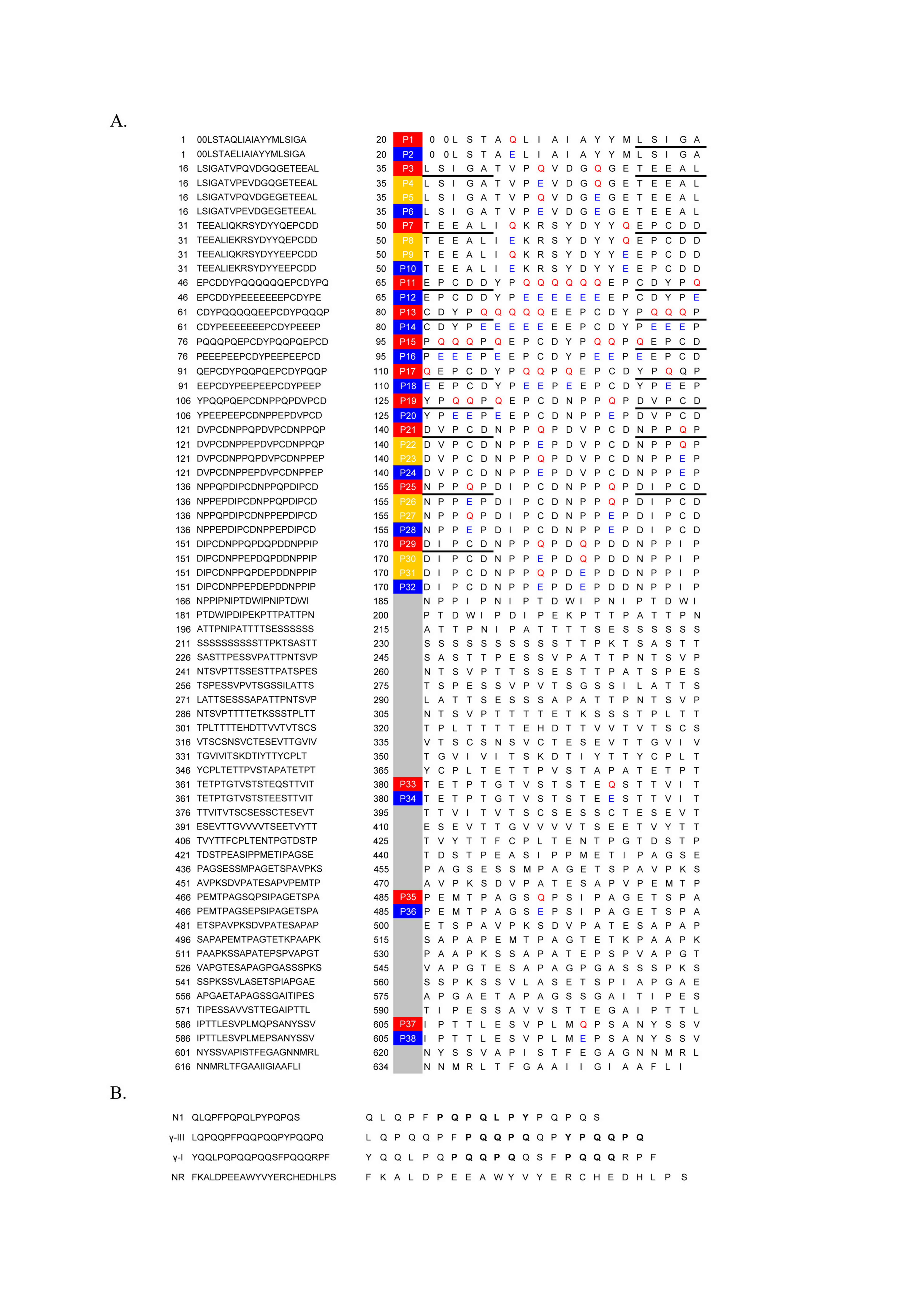

Supplement: S3 Fig — Native peptide (red); peptides with a switch of glutamine to glutamic acid in order to mimic the enzymic conversion carried out by transglutaminase: all Q changed for E (blue), combinatory changes for one Q/E replacement (yellow). Gray represents peptides not synthesized. Overlaps are underlined in black. (B) Sequences of the 17–23-mer peptides designed from different forms of gliadin (N1, γ I, γ III) and non-relevant peptide (NR). Homology motifs between Hwp1 and gliadin are in bold (PQQPQ: 3 and 5 repeating motif, respectively, in γ-gliadin and Hwp1; YPQQPQ, PQQQ: common motifs in γ-gliadin and Hwp1; PQPQLPY: α-gliadin motif having high sequence homology with the repeated sequence PQPDIPC in Hwp1). (TIF) [file pone.0121776.s003.tif]
