# Supplementary material for: Humoral Immunity Links Candida albicans Infection and Celiac Disease
Source: PLoS One. 2015 Mar 20;10(3):e0121776. doi: 10.1371/journal.pone.0121776 (PMC4368562; doi:10.1371/journal.pone.0121776)
Supplement: S1 Materials and Methods — (DOCX) [file pone.0121776.s004.docx]

**Supplementary Materials and Methods :**

***A. Cloning, expression and purification of rNtermHwp1***

*E. coli* DE3 cells expressing rNTerm Hwp1[[26](#_ENREF_26), [29](#_ENREF_29)] were grown overnight in LB broth containing ampicillin (100 µg/mL) (Sigma) at 37°C. The culture was used as the inoculum for fresh LB medium containing ampicillin and was incubated until an OD_600_ of 0.8 was reached. Production of rNtermHwp1 was then induced for 4 h with 0.5 mM isopropyl-ß-D-thiogalactoside (Sigma). The 6x-His tagged rNtermHwp1 was purified by nickel chelate affinity chromatography in accordance with the manufacturer’s instructions (Sigma), dialyzed against 0.02 M phosphate-buffered saline (PBS) pH 7.4, and stored at -20°C.

***B. SDS-PAGE and western blotting***

Purified rNterm Hwp1 was visualized by SDS-PAGE on 10% (w/v) acrylamide gels. The protein was stained directly in the gel with PageBlue Protein Staining solution (Fermentas) or transferred onto a polyvinylidene difloride membrane (Millipore). Hwp1 was dectected with mAb 16B1. A nickel-nitrilotriacetic acid conjugate (nickel-HRP HisDetector; KPL) was used to detect the reaction, as described previously[[29](#_ENREF_29)].

***C. Peptide arrays***

Peptides were synthesized by solid phase peptide synthesis with an automated peptide synthesizer (Intavis AG, Köln, Germany) using the Fmoc/tert-butyl strategy on a 15 µmol scale on a H-PAL-ChemMatrix® (PCAS BioMatrix Inc, Saint-Jean-sur-Richelieu, Quebec) resin. Following their elongation, peptides were deprotected and cleaved for 2 h at RT using TFA/water/triisopropylsilane (950 µL/25 µL/25 µL), precipitated in diethyl ether/n-heptane, 1/1 v/v, purified by RP-HPLC on a 120 Å 5 µm C18 Nucleosil column using a linear water/acetonitrile gradient containing 0.05 % TFA by vol (6 mL/min, detection at 230 nm) and lyophilized.

Each peptide characterized by RP-HPLC and MALDI-TOF MS was dissolved to a final concentration of 0.1 mM in 0.01 M PBS, pH 7.4, and printed on amine-modified glass slides (Arrayit, Sunnyvale, US) in duplicate.

Peptide arrays were blocked for 1 h at RT with PBS-M (0.01 M PBS, pH7.4, 0.05% Tween 20 and 2.5% non-fat milk). Saturated microarrays were washed with PBS containing 0.05% Tween 20. Human sera were diluted 1:100 in PBS-M and incubated overnight at 4°C. Microarrays were then washed 3 times with PBS containing 0.05% Tween 20. After washing, microarrays were revealed using a fluorescein-labeled goat polyclonal anti-human IgG antibody (Abcam, Cambridge, UK) at 0.2 µg/mL in PBS-M or with a fluorescein-labeled goat polyclonal anti-human IgA antibody (PARIS Anticorps, Compiègne, France) at 1 µg/mL in PBS-M, for 1 h at RT. Microarrays were washed, rinsed with distilled water, and dried. The glass slides were scanned with a TECAN LS-reloaded scanner (Tecan, Männedorf, Switzerland): PMT = 150. Data were extracted using Array-Pro® Analyzer Software.

*Epitope mapping of the Mab 16B1* was performed according the same protocol, the Mab was used at a dilution of 1:400 and its binding revealed by an anti-mouse IgG conjugate at 2mg/mL (PARIS Anticorps, Compiègne, France) diluted 1:5000.
